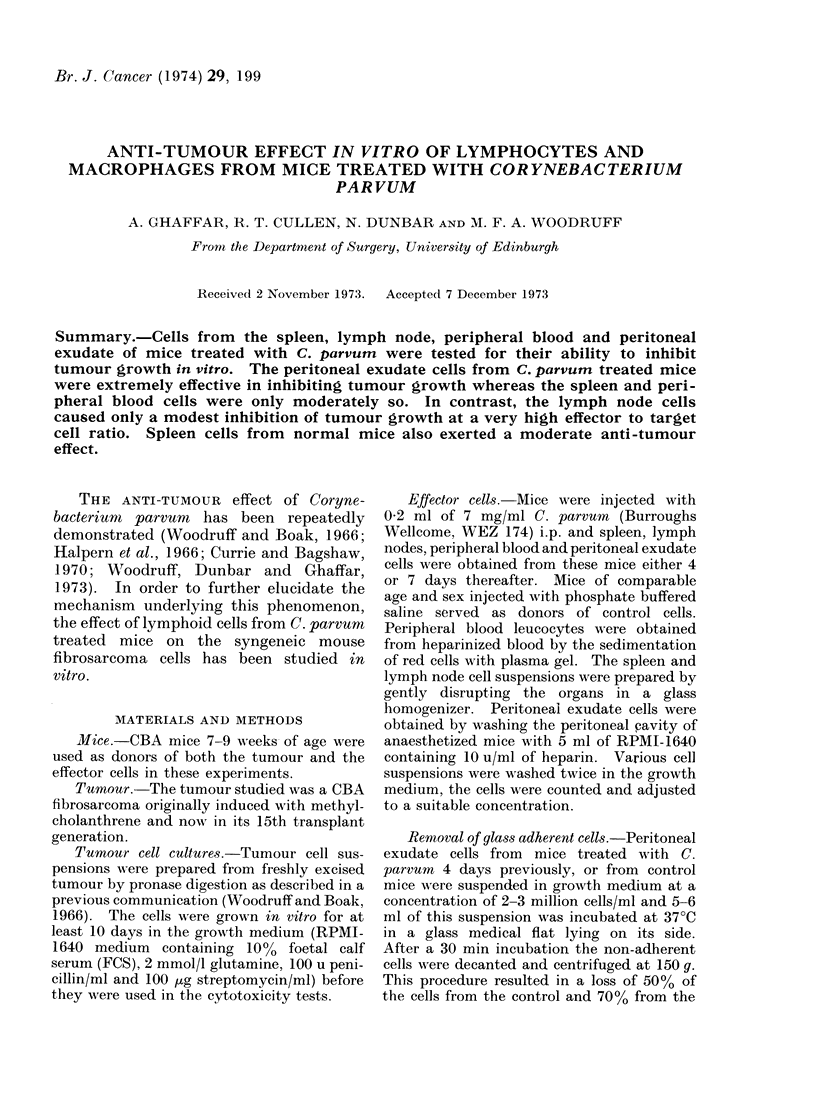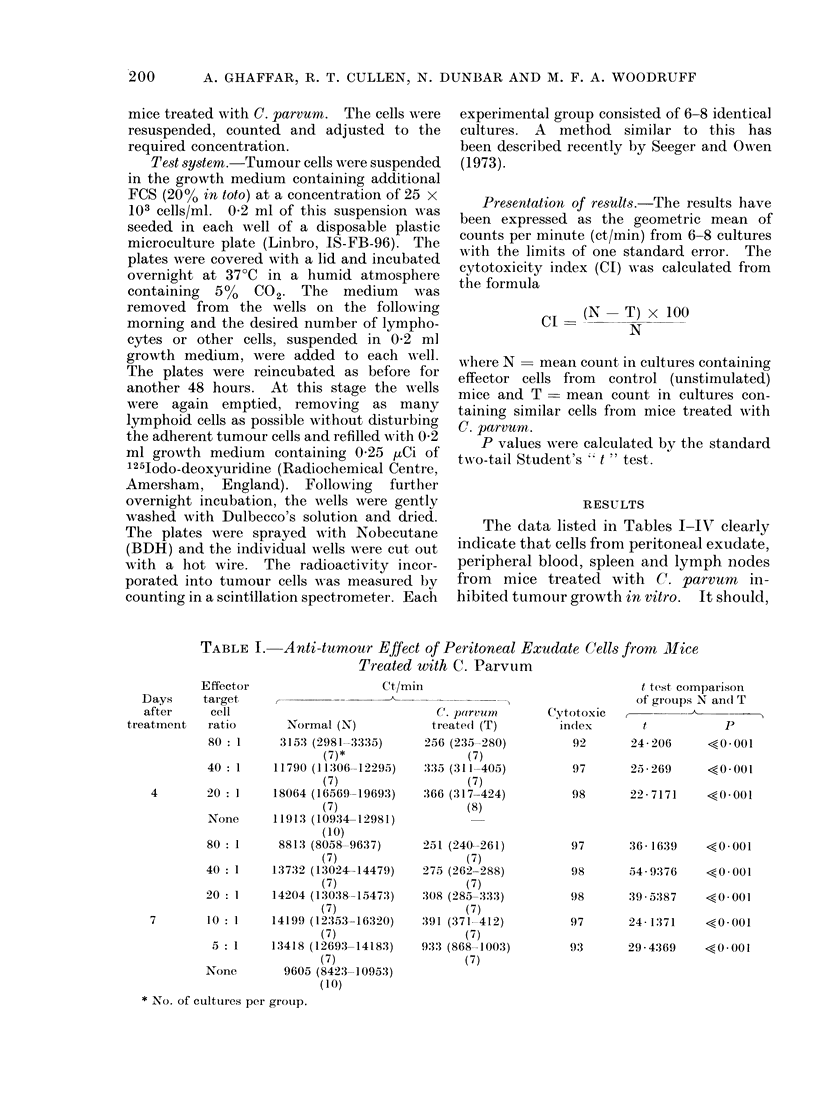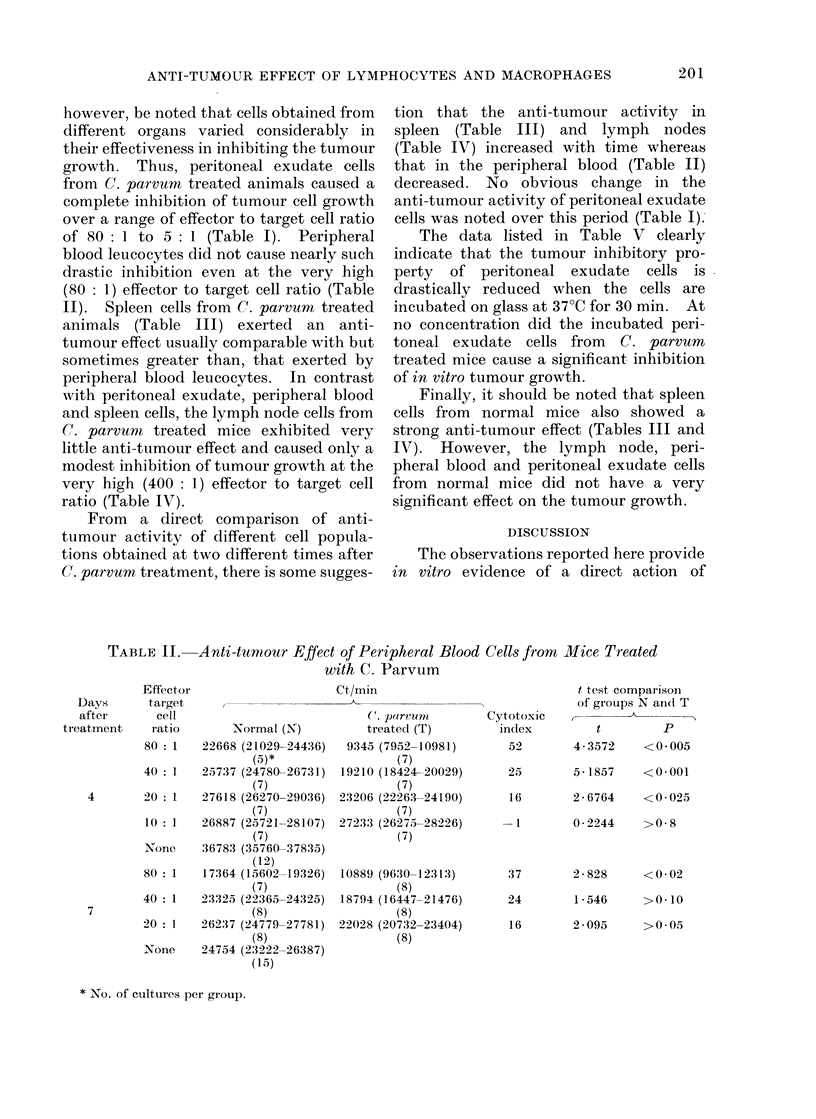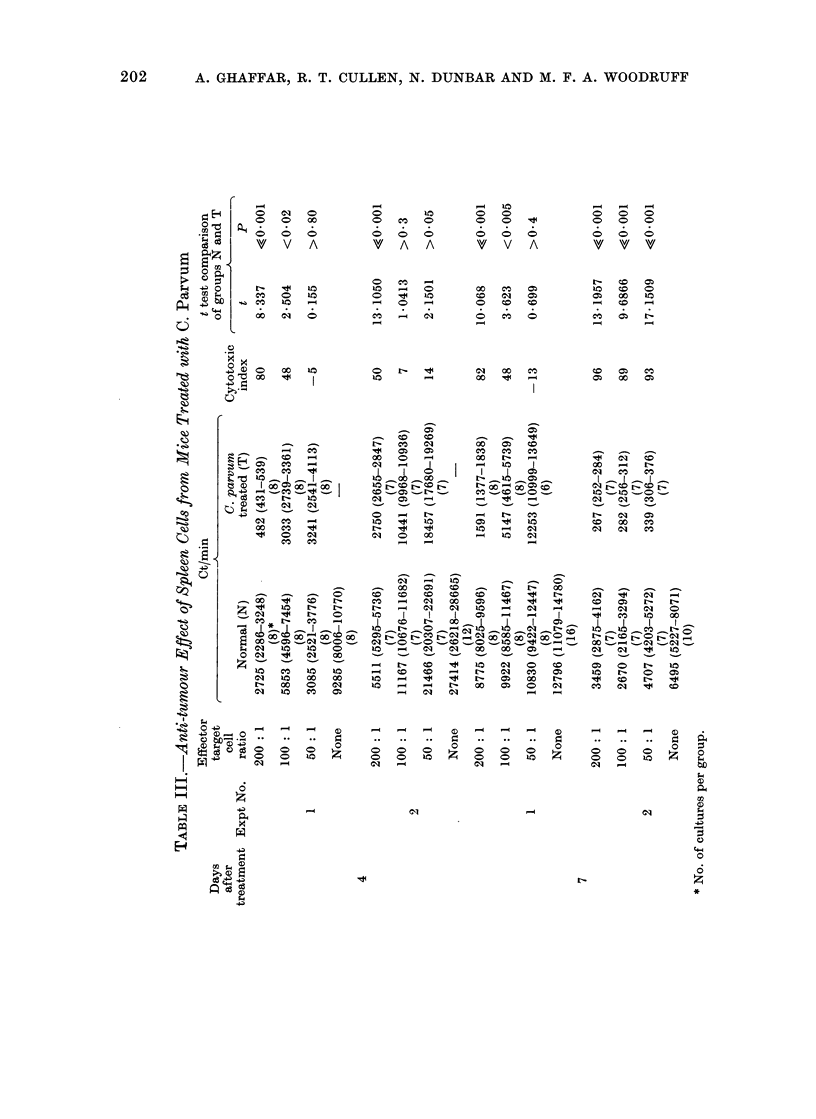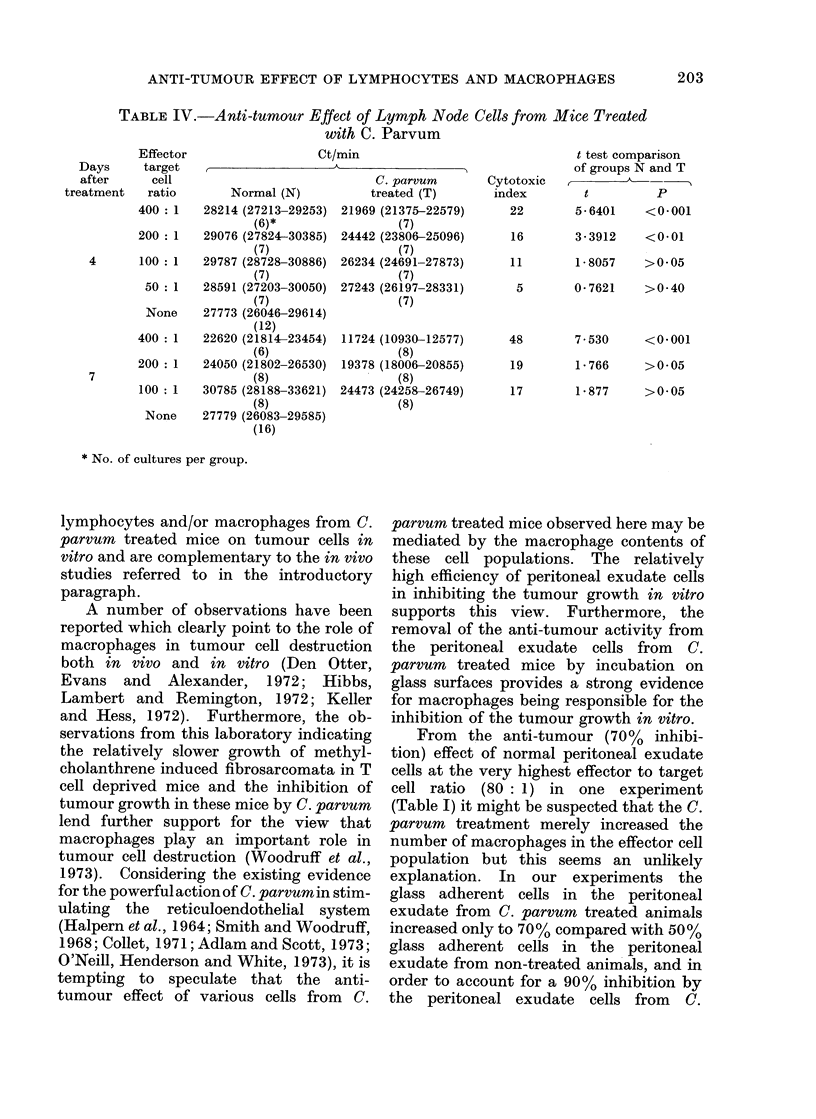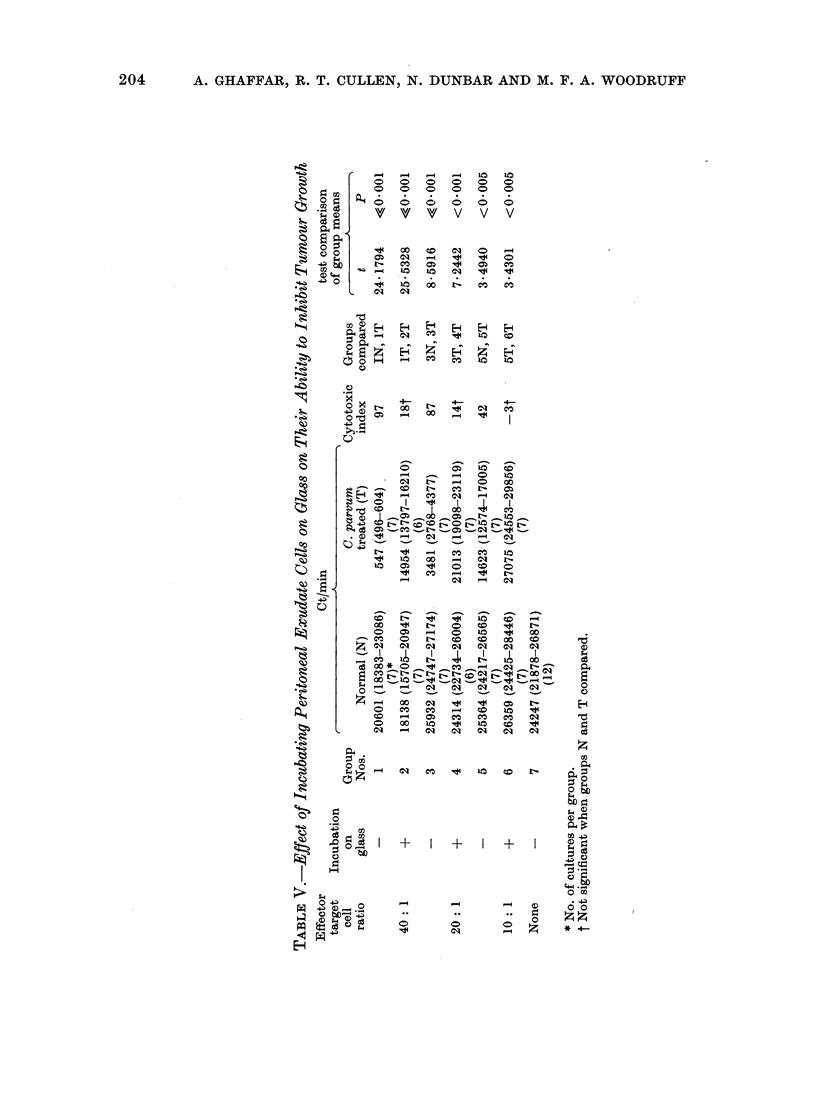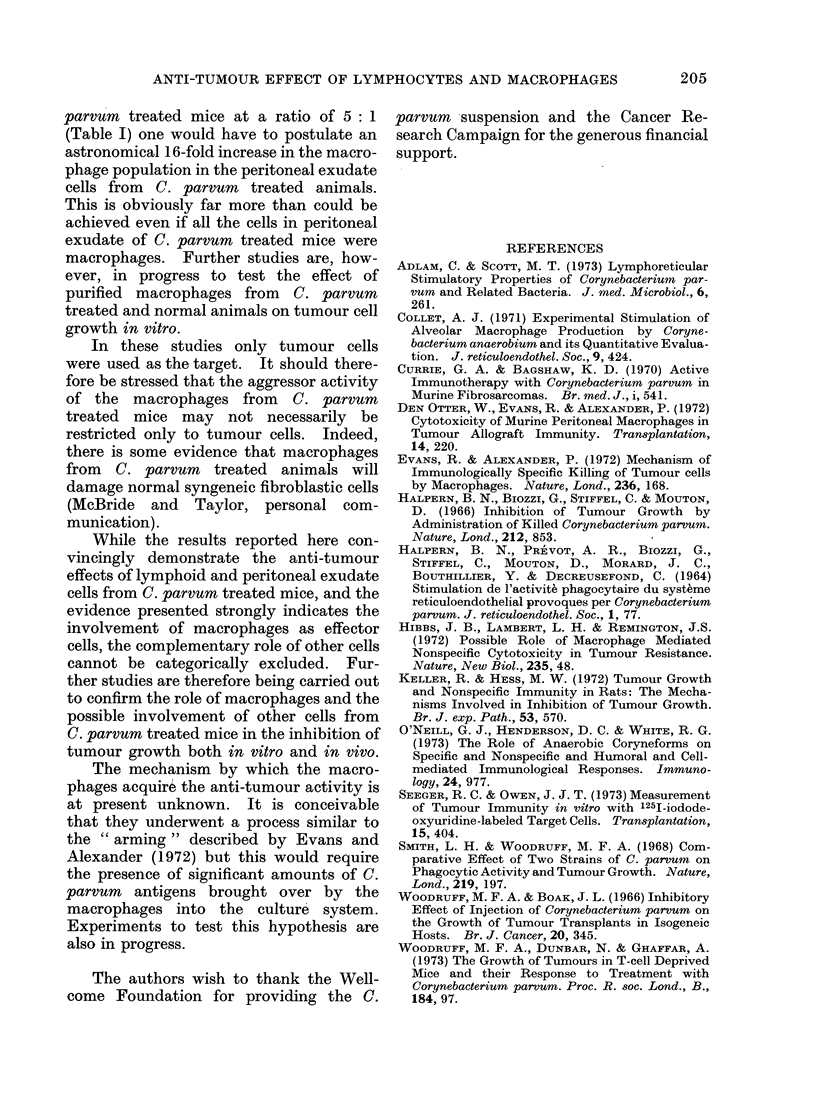# Anti-Tumour Effect In Vitro of Lymphocytes and Macrophages from Mice Treated with Corynebacterium Parvum

**DOI:** 10.1038/bjc.1974.59

**Published:** 1974-03

**Authors:** A. Ghaffar, R. T. Cullen, N. Dunbar, M. F. A. Woodruff

## Abstract

Cells from the spleen, lymph node, peripheral blood and peritoneal exudate of mice treated with *C. parvum* were tested for their ability to inhibit tumour growth *in vitro.* The peritoneal exudate cells from *C. parvum* treated mice were extremely effective in inhibiting tumour growth whereas the spleen and peripheral blood cells were only moderately so. In contrast, the lymph node cells caused only a modest inhibition of tumour growth at a very high effector to target cell ratio. Spleen cells from normal mice also exerted a moderate anti-tumour effect.


*Br. J. Cancer* (1974) **29**, 199

### ANTI-TUMOUR EFFECT *IN VITRO* OF LYMPHOCYTES AND MACROPHAGES FROM MICE TREATED WITH *CORYNEBACTERIUM PARVUM*

A. GHAFFAR, R. T. CULLEN, N. DUNBAR AND M. F. A. WOODRUFF

*From the Department of Surgery, University of Edinburgh*

Received 2 November 1973. Accepted 7 December 1973

**Summary.—Cells from the spleen, lymph node, peripheral blood and peritoneal exudate of mice treated with *C. parvum* were tested for their ability to inhibit tumour growth *in vitro*. The peritoneal exudate cells from *C. parvum* treated mice were extremely effective in inhibiting tumour growth whereas the spleen and peripheral blood cells were only moderately so. In contrast, the lymph node cells caused only a modest inhibition of tumour growth at a very high effector to target cell ratio. Spleen cells from normal mice also exerted a moderate anti-tumour effect.**

THE ANTI-TUMOUR effect of *Corynebacterium parvum* has been repeatedly demonstrated (Woodruff and Boak, 1966; Halpern *et al.*, 1966; Currie and Bagshaw, 1970; Woodruff, Dunbar and Ghaffar, 1973). In order to further elucidate the mechanism underlying this phenomenon, the effect of lymphoid cells from *C. parvum* treated mice on the syngeneic mouse fibrosarcoma cells has been studied *in vitro*.

### MATERIALS AND METHODS

*Mice.*—CBA mice 7–9 weeks of age were used as donors of both the tumour and the effector cells in these experiments.

*Tumour.*—The tumour studied was a CBA fibrosarcoma originally induced with methylcholanthrene and now in its 15th transplant generation.

*Tumour cell cultures.*—Tumour cell suspensions were prepared from freshly excised tumour by pronase digestion as described in a previous communication (Woodruff and Boak, 1966). The cells were grown *in vitro* for at least 10 days in the growth medium (RPMI-1640 medium containing 10% foetal calf serum (FCS), 2 mmol/l glutamine, 100 u penicillin/ml and 100 μg streptomycin/ml) before they were used in the cytotoxicity tests.

*Effector cells.*—Mice were injected with 0·2 ml of 7 mg/ml *C. parvum* (Burroughs Wellcome, WEZ 174) i.p. and spleen, lymph nodes, peripheral blood and peritoneal exudate cells were obtained from these mice either 4 or 7 days thereafter. Mice of comparable age and sex injected with phosphate buffered saline served as donors of control cells. Peripheral blood leucocytes were obtained from heparinized blood by the sedimentation of red cells with plasma gel. The spleen and lymph node cell suspensions were prepared by gently disrupting the organs in a glass homogenizer. Peritoneal exudate cells were obtained by washing the peritoneal cavity of anaesthetized mice with 5 ml of RPMI-1640 containing 10 u/ml of heparin. Various cell suspensions were washed twice in the growth medium, the cells were counted and adjusted to a suitable concentration.

*Removal of glass adherent cells.*—Peritoneal exudate cells from mice treated with *C. parvum* 4 days previously, or from control mice were suspended in growth medium at a concentration of 2–3 million cells/ml and 5–6 ml of this suspension was incubated at 37°C in a glass medical flat lying on its side. After a 30 min incubation the non-adherent cells were decanted and centrifuged at 150 *g*. This procedure resulted in a loss of 50% of the cells from the control and 70% from the mice treated with *C. parvum*. The cells were resuspended, counted and adjusted to the required concentration.

*Test system.*—Tumour cells were suspended in the growth medium containing additional FCS (20% *in toto*) at a concentration of $25 \times 10^3$ cells/ml. 0·2 ml of this suspension was seeded in each well of a disposable plastic microculture plate (Linbro, IS-FB-96). The plates were covered with a lid and incubated overnight at 37°C in a humid atmosphere containing 5% $CO_2$. The medium was removed from the wells on the following morning and the desired number of lymphocytes or other cells, suspended in 0·2 ml growth medium, were added to each well. The plates were reincubated as before for another 48 hours. At this stage the wells were again emptied, removing as many lymphoid cells as possible without disturbing the adherent tumour cells and refilled with 0·2 ml growth medium containing 0·25 μCi of $^{125}$Iodo-deoxyuridine (Radiochemical Centre, Amersham, England). Following further overnight incubation, the wells were gently washed with Dulbecco's solution and dried. The plates were sprayed with Nobecutane (BDH) and the individual wells were cut out with a hot wire. The radioactivity incorporated into tumour cells was measured by counting in a scintillation spectrometer. Each experimental group consisted of 6–8 identical cultures. A method similar to this has been described recently by Seeger and Owen (1973).

*Presentation of results.*—The results have been expressed as the geometric mean of counts per minute (ct/min) from 6–8 cultures with the limits of one standard error. The cytotoxicity index (CI) was calculated from the formula

$$CI = \frac{(N - T) \times 100}{N}$$

where N = mean count in cultures containing effector cells from control (unstimulated) mice and T = mean count in cultures containing similar cells from mice treated with *C. parvum*.

*P* values were calculated by the standard two-tail Student's "*t*" test.

### RESULTS

The data listed in Tables I–IV clearly indicate that cells from peritoneal exudate, peripheral blood, spleen and lymph nodes from mice treated with *C. parvum* inhibited tumour growth *in vitro*. It should,

TABLE I.—*Anti-tumour Effect of Peritoneal Exudate Cells from Mice Treated with* C. Parvum

| Days after treatment | Effector target cell ratio | Ct/min: Normal (N) | Ct/min: *C. parvum* treated (T) | Cytotoxic index | *t* test comparison of groups N and T: *t* | *P* |
|---|---|---|---|---|---|---|
| | 80 : 1 | 3153 (2981–3335) (7)* | 256 (235–280) (7) | 92 | 24·206 | ⩽0·001 |
| | 40 : 1 | 11790 (11306–12295) (7) | 335 (311–405) (7) | 97 | 25·269 | ⩽0·001 |
| 4 | 20 : 1 | 18064 (16569–19693) (7) | 366 (317–424) (8) | 98 | 22·7171 | ⩽0·001 |
| | None | 11913 (10934–12981) (10) | — | | | |
| | 80 : 1 | 8813 (8058–9637) (7) | 251 (240–261) (7) | 97 | 36·1639 | ⩽0·001 |
| | 40 : 1 | 13732 (13024–14479) (7) | 275 (262–288) (7) | 98 | 54·9376 | ⩽0·001 |
| | 20 : 1 | 14204 (13038–15473) (7) | 308 (285–333) (7) | 98 | 39·5387 | ⩽0·001 |
| 7 | 10 : 1 | 14199 (12353–16320) (7) | 391 (371–412) (7) | 97 | 24·1371 | ⩽0·001 |
| | 5 : 1 | 13418 (12693–14183) (7) | 933 (868–1003) (7) | 93 | 29·4369 | ⩽0·001 |
| | None | 9605 (8423–10953) (10) | | | | |

* No. of cultures per group.

however, be noted that cells obtained from different organs varied considerably in their effectiveness in inhibiting the tumour growth. Thus, peritoneal exudate cells from *C. parvum* treated animals caused a complete inhibition of tumour cell growth over a range of effector to target cell ratio of 80 : 1 to 5 : 1 (Table I). Peripheral blood leucocytes did not cause nearly such drastic inhibition even at the very high (80 : 1) effector to target cell ratio (Table II). Spleen cells from *C. parvum* treated animals (Table III) exerted an anti-tumour effect usually comparable with but sometimes greater than, that exerted by peripheral blood leucocytes. In contrast with peritoneal exudate, peripheral blood and spleen cells, the lymph node cells from *C. parvum* treated mice exhibited very little anti-tumour effect and caused only a modest inhibition of tumour growth at the very high (400 : 1) effector to target cell ratio (Table IV).

From a direct comparison of anti-tumour activity of different cell populations obtained at two different times after *C. parvum* treatment, there is some suggestion that the anti-tumour activity in spleen (Table III) and lymph nodes (Table IV) increased with time whereas that in the peripheral blood (Table II) decreased. No obvious change in the anti-tumour activity of peritoneal exudate cells was noted over this period (Table I).

The data listed in Table V clearly indicate that the tumour inhibitory property of peritoneal exudate cells is drastically reduced when the cells are incubated on glass at 37°C for 30 min. At no concentration did the incubated peritoneal exudate cells from *C. parvum* treated mice cause a significant inhibition of *in vitro* tumour growth.

Finally, it should be noted that spleen cells from normal mice also showed a strong anti-tumour effect (Tables III and IV). However, the lymph node, peripheral blood and peritoneal exudate cells from normal mice did not have a very significant effect on the tumour growth.

### DISCUSSION

The observations reported here provide *in vitro* evidence of a direct action of

TABLE II.—*Anti-tumour Effect of Peripheral Blood Cells from Mice Treated with* C. Parvum

| Days after treatment | Effector target cell ratio | Ct/min: Normal (N) | Ct/min: *C. parvum* treated (T) | Cytotoxic index | t test comparison of groups N and T: *t* | *P* |
|---|---|---|---|---|---|---|
| 4 | 80 : 1 | 22668 (21029–24436) (5)* | 9345 (7952–10981) (7) | 52 | 4·3572 | <0·005 |
| | 40 : 1 | 25737 (24780–26731) (7) | 19210 (18424–20029) (7) | 25 | 5·1857 | <0·001 |
| | 20 : 1 | 27618 (26270–29036) (7) | 23206 (22263–24190) (7) | 16 | 2·6764 | <0·025 |
| | 10 : 1 | 26887 (25721–28107) (7) | 27233 (26275–28226) (7) | −1 | 0·2244 | >0·8 |
| | None | 36783 (35760–37835) (12) | | | | |
| 7 | 80 : 1 | 17364 (15602–19326) (7) | 10889 (9630–12313) (8) | 37 | 2·828 | <0·02 |
| | 40 : 1 | 23325 (22365–24325) (8) | 18794 (16447–21476) (8) | 24 | 1·546 | >0·10 |
| | 20 : 1 | 26237 (24779–27781) (8) | 22028 (20732–23404) (8) | 16 | 2·095 | >0·05 |
| | None | 24754 (23222–26387) (15) | | | | |

* No. of cultures per group.

TABLE III.—*Anti-tumour Effect of Spleen Cells from Mice Treated with* C. Parvum

| Days after treatment | Expt No. | Effector target cell ratio | Ct/min Normal (N) | Ct/min *C. parvum* treated (T) | Cytotoxic index | *t* test comparison of groups N and T: *t* | *t* test comparison of groups N and T: *P* |
|---|---|---|---|---|---|---|---|
| 4 | 1 | 200 : 1 | 2725 (2286–3248) (8)* | 482 (431–539) (8) | 80 | 8·337 | ⩽0·001 |
| | | 100 : 1 | 5853 (4596–7454) (8) | 3033 (2739–3361) (8) | 48 | 2·504 | <0·02 |
| | | 50 : 1 | 3085 (2521–3776) (8) | 3241 (2541–4113) (8) | −5 | 0·155 | >0·80 |
| | | None | 9285 (8006–10770) (8) | — | | | |
| | 2 | 200 : 1 | 5511 (5295–5736) (7) | 2750 (2655–2847) (7) | 50 | 13·1050 | ⩽0·001 |
| | | 100 : 1 | 11167 (10676–11682) (7) | 10441 (9968–10936) (7) | 7 | 1·0413 | >0·3 |
| | | 50 : 1 | 21466 (20307–22691) (7) | 18457 (17680–19269) (7) | 14 | 2·1501 | >0·05 |
| | | None | 27414 (26218–28665) (12) | — | | | |
| 7 | 1 | 200 : 1 | 8775 (8025–9596) (8) | 1591 (1377–1838) (8) | 82 | 10·068 | ⩽0·001 |
| | | 100 : 1 | 9922 (8585–11467) (8) | 5147 (4615–5739) (8) | 48 | 3·623 | <0·005 |
| | | 50 : 1 | 10830 (9422–12447) (8) | 12253 (10999–13649) (6) | −13 | 0·699 | >0·4 |
| | | None | 12796 (11079–14780) (16) | | | | |
| | 2 | 200 : 1 | 3459 (2875–4162) (7) | 267 (252–284) (7) | 96 | 13·1957 | ⩽0·001 |
| | | 100 : 1 | 2670 (2165–3294) (7) | 282 (256–312) (7) | 89 | 9·6866 | ⩽0·001 |
| | | 50 : 1 | 4707 (4203–5272) (7) | 339 (306–376) (7) | 93 | 17·1509 | ⩽0·001 |
| | | None | 6495 (5227–8071) (10) | | | | |

* No. of cultures per group.

TABLE IV.—*Anti-tumour Effect of Lymph Node Cells from Mice Treated with* C. Parvum

| Days after treatment | Effector target cell ratio | Ct/min Normal (N) | Ct/min *C. parvum* treated (T) | Cytotoxic index | *t* test comparison of groups N and T: *t* | *P* |
|---|---|---|---|---|---|---|
| 4 | 400 : 1 | 28214 (27213–29253) (6)* | 21969 (21375–22579) (7) | 22 | 5·6401 | <0·001 |
| | 200 : 1 | 29076 (27824–30385) (7) | 24442 (23806–25096) (7) | 16 | 3·3912 | <0·01 |
| | 100 : 1 | 29787 (28728–30886) (7) | 26234 (24691–27873) (7) | 11 | 1·8057 | >0·05 |
| | 50 : 1 | 28591 (27203–30050) (7) | 27243 (26197–28331) (7) | 5 | 0·7621 | >0·40 |
| | None | 27773 (26046–29614) (12) | | | | |
| 7 | 400 : 1 | 22620 (21814–23454) (6) | 11724 (10930–12577) (8) | 48 | 7·530 | <0·001 |
| | 200 : 1 | 24050 (21802–26530) (8) | 19378 (18006–20855) (8) | 19 | 1·766 | >0·05 |
| | 100 : 1 | 30785 (28188–33621) (8) | 24473 (24258–26749) (8) | 17 | 1·877 | >0·05 |
| | None | 27779 (26083–29585) (16) | | | | |

* No. of cultures per group.

lymphocytes and/or macrophages from *C. parvum* treated mice on tumour cells *in vitro* and are complementary to the *in vivo* studies referred to in the introductory paragraph.

A number of observations have been reported which clearly point to the role of macrophages in tumour cell destruction both *in vivo* and *in vitro* (Den Otter, Evans and Alexander, 1972; Hibbs, Lambert and Remington, 1972; Keller and Hess, 1972). Furthermore, the observations from this laboratory indicating the relatively slower growth of methylcholanthrene induced fibrosarcomata in T cell deprived mice and the inhibition of tumour growth in these mice by *C. parvum* lend further support for the view that macrophages play an important role in tumour cell destruction (Woodruff *et al.*, 1973). Considering the existing evidence for the powerful action of *C. parvum* in stimulating the reticuloendothelial system (Halpern *et al.*, 1964; Smith and Woodruff, 1968; Collet, 1971; Adlam and Scott, 1973; O'Neill, Henderson and White, 1973), it is tempting to speculate that the anti-tumour effect of various cells from *C. parvum* treated mice observed here may be mediated by the macrophage contents of these cell populations. The relatively high efficiency of peritoneal exudate cells in inhibiting the tumour growth *in vitro* supports this view. Furthermore, the removal of the anti-tumour activity from the peritoneal exudate cells from *C. parvum* treated mice by incubation on glass surfaces provides a strong evidence for macrophages being responsible for the inhibition of the tumour growth *in vitro.*

From the anti-tumour (70% inhibition) effect of normal peritoneal exudate cells at the very highest effector to target cell ratio (80 : 1) in one experiment (Table I) it might be suspected that the *C. parvum* treatment merely increased the number of macrophages in the effector cell population but this seems an unlikely explanation. In our experiments the glass adherent cells in the peritoneal exudate from *C. parvum* treated animals increased only to 70% compared with 50% glass adherent cells in the peritoneal exudate from non-treated animals, and in order to account for a 90% inhibition by the peritoneal exudate cells from *C.*

TABLE V.—*Effect of Incubating Peritoneal Exudate Cells on Glass on Their Ability to Inhibit Tumour Growth*

| Effector target cell ratio | Incubation on glass | Group Nos. | Ct/min | | Cytotoxic index | Groups compared | test comparison of group means | |
|---|---|---|---|---|---|---|---|---|
| | | | Normal (N) | *C. parvum* treated (T) | | | $t$ | $P$ |
| | − | 1 | 20601 (18383–23086) (7)* | 547 (496–604) (7) | 97 | IN, 1T | 24·1794 | ⩽0·001 |
| 40 : 1 | + | 2 | 18138 (15705–20947) (7) | 14954 (13797–16210) (6) | 18† | 1T, 2T | 25·5328 | ⩽0·001 |
| | − | 3 | 25932 (24747–27174) (7) | 3481 (2768–4377) (7) | 87 | 3N, 3T | 8·5916 | ⩽0·001 |
| 20 : 1 | + | 4 | 24314 (22734–26004) (6) | 21013 (19098–23119) (7) | 14† | 3T, 4T | 7·2442 | <0·001 |
| | − | 5 | 25364 (24217–26565) (7) | 14623 (12574–17005) (7) | 42 | 5N, 5T | 3·4940 | <0·005 |
| 10 : 1 | + | 6 | 26359 (24425–28446) (7) | 27075 (24553–29856) (7) | −3† | 5T, 6T | 3·4301 | <0·005 |
| None | − | 7 | 24247 (21878–26871) (12) | | | | | |

* No. of cultures per group.

† Not significant when groups N and T compared.

*parvum* treated mice at a ratio of 5 : 1 (Table I) one would have to postulate an astronomical 16-fold increase in the macrophage population in the peritoneal exudate cells from *C. parvum* treated animals. This is obviously far more than could be achieved even if all the cells in peritoneal exudate of *C. parvum* treated mice were macrophages. Further studies are, however, in progress to test the effect of purified macrophages from *C. parvum* treated and normal animals on tumour cell growth *in vitro*.

In these studies only tumour cells were used as the target. It should therefore be stressed that the aggressor activity of the macrophages from *C. parvum* treated mice may not necessarily be restricted only to tumour cells. Indeed, there is some evidence that macrophages from *C. parvum* treated animals will damage normal syngeneic fibroblastic cells (McBride and Taylor, personal communication).

While the results reported here convincingly demonstrate the anti-tumour effects of lymphoid and peritoneal exudate cells from *C. parvum* treated mice, and the evidence presented strongly indicates the involvement of macrophages as effector cells, the complementary role of other cells cannot be categorically excluded. Further studies are therefore being carried out to confirm the role of macrophages and the possible involvement of other cells from *C. parvum* treated mice in the inhibition of tumour growth both *in vitro* and *in vivo*.

The mechanism by which the macrophages acquire the anti-tumour activity is at present unknown. It is conceivable that they underwent a process similar to the "arming" described by Evans and Alexander (1972) but this would require the presence of significant amounts of *C. parvum* antigens brought over by the macrophages into the culture system. Experiments to test this hypothesis are also in progress.

The authors wish to thank the Wellcome Foundation for providing the *C. parvum* suspension and the Cancer Research Campaign for the generous financial support.

### REFERENCES

ADLAM, C. & SCOTT, M. T. (1973) Lymphoreticular Stimulatory Properties of *Corynebacterium parvum* and Related Bacteria. *J. med. Microbiol.*, **6**, 261.

COLLET, A. J. (1971) Experimental Stimulation of Alveolar Macrophage Production by *Corynebacterium anaerobium* and its Quantitative Evaluation. *J. reticuloendothel. Soc.*, **9**, 424.

CURRIE, G. A. & BAGSHAW, K. D. (1970) Active Immunotherapy with *Corynebacterium parvum* in Murine Fibrosarcomas. *Br. med. J.*, i, 541.

DEN OTTER, W., EVANS, R. & ALEXANDER, P. (1972) Cytotoxicity of Murine Peritoneal Macrophages in Tumour Allograft Immunity. *Transplantation*, **14**, 220.

EVANS, R. & ALEXANDER, P. (1972) Mechanism of Immunologically Specific Killing of Tumour cells by Macrophages. *Nature, Lond.*, **236**, 168.

HALPERN, B. N., BIOZZI, G., STIFFEL, C. & MOUTON, D. (1966) Inhibition of Tumour Growth by Administration of Killed *Corynebacterium parvum*. *Nature, Lond.*, **212**, 853.

HALPERN, B. N., PRÉVOT, A. R., BIOZZI, G., STIFFEL, C., MOUTON, D., MORARD, J. C., BOUTHILLIER, Y. & DECREUSEFOND, C. (1964) Stimulation de l'activitè phagocytaire du systême reticuloendothelial provoques per *Corynebacterium parvum*. *J. reticuloendothel. Soc.*, **1**, 77.

HIBBS, J. B., LAMBERT, L. H. & REMINGTON, J.S. (1972) Possible Role of Macrophage Mediated Nonspecific Cytotoxicity in Tumour Resistance. *Nature, New Biol.*, **235**, 48.

KELLER, R. & HESS, M. W. (1972) Tumour Growth and Nonspecific Immunity in Rats: The Mechanisms Involved in Inhibition of Tumour Growth. *Br. J. exp. Path.*, **53**, 570.

O'NEILL, G. J., HENDERSON, D. C. & WHITE, R. G. (1973) The Role of Anaerobic Coryneforms on Specific and Nonspecific and Humoral and Cell-mediated Immunological Responses. *Immunology*, **24**, 977.

SEEGER, R. C. & OWEN, J. J. T. (1973) Measurement of Tumour Immunity *in vitro* with $^{125}$I-iododeoxyuridine-labeled Target Cells. *Transplantation*, **15**, 404.

SMITH, L. H. & WOODRUFF, M. F. A. (1968) Comparative Effect of Two Strains of *C. parvum* on Phagocytic Activity and Tumour Growth. *Nature, Lond.*, **219**, 197.

WOODRUFF, M. F. A. & BOAK, J. L. (1966) Inhibitory Effect of Injection of *Corynebacterium parvum* on the Growth of Tumour Transplants in Isogeneic Hosts. *Br. J. Cancer*, **20**, 345.

WOODRUFF, M. F. A., DUNBAR, N. & GHAFFAR, A. (1973) The Growth of Tumours in T-cell Deprived Mice and their Response to Treatment with *Corynebacterium parvum*. *Proc. R. soc. Lond., B.*, **184**, 97.